# Wasted Opportunities

**Published:** 1890-08-30

**Authors:** 


### Words of Consolation.

### WASTED OPPORTUNITIES.

If we had only known! What a vista of lost opportunities and neglected duties rise up before us at these words. If we had only known how they would have ended, how different our actions would have been! At the outset of life we meant to have made our mark in the world, to have startled men in some way, by our learning, or our talents in making a large fortune, or even our goodness perhaps; but when the sands of time begin to run swiftly, and we are nearing the end of our course, can we venture to look back on our career, and if so, how does it strike us? Have we done all we hoped to do, or has there been a miserable failure?

In sickness such thoughts as these press on us with an overwhelming force. If I had not been so fond of pleasure says one, I should have worked harder when I was young and strong and been in comfortable circumstances now, instead of dependent on my relations and friends. If I had only taken advice, says another, and not worked so hard, my strength would have lasted, and I should now be able to enjoy the rest and ease which my money can give me, but which alas are useless. A neglected cold has brought on this consumption, says a third; why was I not careful? If I had only known my dear mother would have been taken so soon, I would have tried to please her more and not myself, says a fourth, with a bitterness of spirit which time alone can soften.

And how about the still more important retrospect of our behaviour to our God? Have we any of us been sufficiently grateful to Him for our Creation, for our Redemption, for our preservation and all the blessings of this life? Have we any of us taken His warnings of weakened health or mind, to pull ourselves together and ask ourselves, why and from whence comes this stroke, this cruel disease, this creeping palsy?

Our Lord as He sat on the Mount of Olives wept over the beautiful city of Jerusalem, whose wasted opportunities were about to bring destruction on that favoured city and exclaimed, "If thou hadst known, even thou, at least in this thy day the things which belong unto thy peace! but now they are hid from thine eyes."

The Jews had been chosen by God from all the nations of the earth to be His Peculiar People, yet they slighted His Covenant, disobeyed His laws, murmured in the Wilderness, longed after the Gods of the Heathen, and later on slew His prophets, and were now about to crucify the Lord of Glory.

And are we Christians God's chosen people now, any better than they? Do we never repine at His will, which keeps us in a station of life hateful to us? Do we not long after the flesh-pots of Egypt and the melons and the cucumbers, and the other unattainable delights? Do we set at naught His commandments and make to ourselves gods, such as health, wealth, intellect, or our own stubborn hearts? Too many of us have done so, and as death draws nigh we fear lest we too have outlived our days, and that the things which should be for our peace are hid from our eyes. Happily for us our day is not yet over; the Fountain for sin is still open; while there is life there is hope. Only we must not delay. If in health we did not love God nor strive to serve Him, we must in humble faith and penitence turn to that Saviour who will help us in our utmost need. Our dear Lord will comfort the broken hearted, heal the sick soul, and bring the impatient will into perfect submission to our Heavenly Father's will. Come then at once, lose no time. Life is short, Eternity is long. It is unwise to put off the most important business. It is sinful to withold from God the obedience due to Him. It is dangerous, as are all delays. The convenient season *may* never come to us in health any more than it came to Felix, and it *cannot* come to us in death.

Work out your salvation, then, in fear and trembling while it is still day, for the night cometh when no man can work. Gather up the fragments of time, health, talents, opportunities that are still left; let nothing be wasted. But if you are past all active exertion of mind and body, lay hold of Christ's merits and humbly trust in them; believe firmly that He alone can save, and though you may be lying impotent by the Pool of Bethesda, hopeless of any help from Man or Angel, the Blessed Lord sees you and knows all your case, He will dip you in His Cleansing Blood, and though your sins be as scarlet they shall be white as snow, and though they be like purple, yet they shall be made white as wool.

